# A Combined Screening Platform for HIV Treatment Failure and Resistance

**DOI:** 10.1371/journal.pone.0035401

**Published:** 2012-04-26

**Authors:** Myres W. Tilghman, Susanne May, Josué Pérez-Santiago, Caroline C. Ignacio, Susan J. Little, Douglas D. Richman, Davey M. Smith

**Affiliations:** 1 Department of Medicine, University of California San Diego, La Jolla, California, United States of America; 2 Veterans Affairs San Diego Healthcare System, San Diego, California, United States of America; 3 Department of Biostatistics, University of Washington, Seattle, Washington, United States of America; 4 Department of Pathology, University of California San Diego, La Jolla, California, United States of America; 5 Department of Bioinformatics and Systems Biology, University of California San Diego, La Jolla, California, United States of America; University of Pittsburgh, United States of America

## Abstract

**Background:**

To develop a low cost method to screen for virologic failure of antiretroviral therapy (ART) and HIV-1 drug resistance, we performed a retrospective evaluation of a screening assay using serial dilutions of HIV-1 RNA-spiked blood plasma and samples from patients receiving >6 months of first-line ART.

**Methods:**

Serial dilution testing was used to assess sensitivity of a simple PCR-based assay (targeted at ≥1,000 HIV RNA copies/mL). We created blood plasma minipools of five samples, extracted HIV RNA from the pools, PCR amplified the reverse transcriptase (RT) coding region of the HIV-1 *pol* gene from extracted RNA, sequenced PCR product of positive pools, and used sequences to determine drug resistance. Sensitivity, specificity, and predictive values were determined for different levels of virologic failure based on maximum viral loads of individual samples within a pool.

**Results:**

Of 295 samples analyzed, 43 (15%) had virologic failure at ≥50 copies/mL (range 50–10,500 copies/mL, four at ≥1,000 copies/mL). The assay demonstrated 100% sensitivity to detect virus from these four samples, requiring only one round of PCR, and 56% and 89% sensitivity to detect samples with ≥50 and ≥500 copies/mL using two rounds. Amplified PCR products of all positive pools were successfully sequenced and 30% harbored ≥1 major resistance mutation. This method would have cost 10% of the combined costs of individual viral load and resistance testing.

**Conclusions:**

We present a novel method that can screen for both virologic failure of first-line ART and drug resistance. The method is much less expensive than current methods, which may offer sustainability in resource-limited settings.

## Introduction

Where available, routine HIV viral load testing is recommended to monitor for virologic failure of antiretroviral therapy (ART) [1]. However, commercial viral load assays are expensive and require sophisticated equipment, technical expertise, and maintenance that are not feasible in many resource-limited settings. To decrease the costs of virologic monitoring in these settings, methods incorporating viral load testing using pooled specimens have been evaluated in statistical simulations and retrospective analyses. These methods, which utilize the quantitative results of pooled and selective individual testing using mathematical formulas to resolve positive pools, have been shown to increase efficiency of virologic monitoring and reduce costs using blood plasma specimens and dried blood and plasma spots, while preserving accuracy to detect levels of viremia as low as 50 HIV RNA copies/mL [2], [3], [4], [5].

The objective of virologic monitoring is to determine if a patient with viremia has failed ART due to drug-resistant virus or if first-line therapy may be salvaged (e.g. by increasing adherence or eliminating interacting medications). While drug resistance is more likely with higher levels of viral replication and low levels of viremia are often due to “blips” [6], [7] that carry no long-term clinical significance, the viral load itself is of limited utility in making this distinction. In other words, the most important clinical information is whether or not the patient harbors drug-resistant virus rather than the actual value of the viral load. Failure without resistance is usually attributable to problems with adherence. In resource abundant settings, this distinction is made by performing drug resistance assays on patient samples with virologic failure. However, in most resource-limited settings, drug resistance testing is even less feasible than viral load monitoring due to cost (US$200–400 per genotyping assay). Therefore, in these settings where second-line treatment options are limited and maximizing first-line therapy is of utmost importance, a low-cost assay designed to allow for detection of virologic failure and evaluation for drug resistance mutations would likely be more useful clinically than assays designed to quantify viral load, which are expensive and do not reliably predict the need for a change in therapy when unaccompanied by drug resistance data.

In order to lower the costs associated with identifying virologic failure and drug resistance in the setting of ART, we designed a platform combining sample pooling with qualitative polymerase chain reaction (PCR) amplification of the reverse transcriptase (RT) coding region of the HIV-1 *pol* gene, and sequencing of the PCR product for detection of drug resistance mutations. Use of pooled rather than individual samples decreased assay sensitivity to a level at which virologic failure was likely (targeting ≥1,000 HIV RNA copies/mL) and drug resistance could be assessed. We hypothesized that this method would be useful to screen for both virologic failure of first-line ART and drug resistance in most resource-limited settings.

## Methods

### Ethics Statement

This study was approved by the UCSD Human Research Protections Program. All participants whose samples were used for this study signed a written informed consent.

### Study population and samples

Testing was performed on pooled patient samples and serial dilutions of HIV RNA-spiked HIV-negative blood plasma. For serial dilution testing, a standard solution obtained from the NIH AIDS Reagent Program with a viral load of 150,000 HIV RNA copies/mL was used. Serial dilutions were prepared by combining this solution with HIV-negative blood plasma to obtain samples with HIV RNA copy numbers of 50, 100, 500, 1,000, and 5,000 copies/mL. These serial dilutions were made in triplicate and designed to simulate minipools (consisting of five samples) containing a single specimen of 250, 500, 2,500, 5,000, and 25,000 copies/mL (and four samples with zero copies/mL).

Blood plasma samples collected from participants in the San Diego Primary HIV Infection Program enrolled between January 1998 and January 2007 were used. These participants had initiated ART with at least three agents for ≥6 months (+/−2 weeks). Each sample had previously undergone individual quantitative viral load testing using the ultra-sensitive Amplicor HIV-1 Monitor viral load assay (Roche Molecular Diagnostics, Pleasanton, California). Technicians performing the assay were blinded to patients and viral load results. Multiple samples from some patients were included because of planned 6-month follow-up visits, but each of these samples was obtained at a different time point.

### Nucleic acid amplification and resistance testing

Starting volumes of 500 microliters were used for the serial dilutions and the sample pools that were constructed by combining 100 microliters of 5 individual patient samples. Testing on minipools of 5 patient samples rather than individual samples was performed to decrease assay sensitivity to detect individual patient samples containing ≥1,000 HIV RNA copies/mL, in addition to the expected cost savings from pooled testing. A pool size of 5 was chosen, since it is less technically complex than larger pool sizes and allows for a sufficient volume of each individual sample to be included in the pool. RNA extraction was performed using the QIAmp Viral RNA Purification Spin Protocol (Qiagen, Valencia, California) with a final elution volume of 50 microliters. Reverse transcription was performed using the Ambion® RETROscript kit (Applied Biosystems, Foster City, California), according to the manufacturer's instructions.

To develop the qualitative pooled RT assay, the PCR protocol used the following hemi-nested primers in the HIV-1 RT coding region, which are specific to regions that are conserved across clades (See **Figures S1, S2 and S3**).

*First round*:

CI-POL1 GGAAGAAATCTGTTGACTCAGATTGG (Forward)

3RT ACCCATCCAAAGGAATGGAGGTTCTTTC (Reverse)

*Second round*:

5RT AAATCCATACAATACTCCAGTATTTGC (Forward)

3RT ACCCATCCAAAGGAATGGAGGTTCTTTC (Reverse)

For the first round of PCR, 10 µL of cDNA product were added to 40 µL of reaction mixture that consisted of 0.5 µL of Taq polymerase (Roche), 5 µL of 10× PCR buffer with magnesium chloride (Roche), 1 µL of 10 mM dNTPs (Roche), 31.5 µL of nuclease-free water, and 1 µL each of the CI-Pol1 and 3RT primers. The 50 µL solution was then heated to 95°C for 2 minutes, followed by 35 cycles at 95°C for 30 seconds, 50°C for 30 seconds, and 72°C for 1 minute and a final heating step at 72°C for 10 minutes. For the second round PCR, 5 µL of the first round PCR product was added to 45 µL of reaction mixture that consisted of the same reagents and cycling conditions as for the first round of PCR, except for a slightly larger volume of nuclease-free water (36.5 µL), and 1 µL each of the 5RT and 3RT primers. One round of PCR was performed on the serial dilutions of RNA in spiked HIV-negative blood plasma, and both rounds of PCR were performed on the minipools. The presence or absence of PCR product was assessed using agarose gel electrophoresis after each round. Although this platform is designed for deconvolution of positive pools by retesting of individual samples included in pools with detectable PCR product, this was not performed for this proof-of-principle experiment due to limited stored sample volumes. The amplified PCR product for each positive pool was then sequenced using a “home brew” Sanger sequencing technique, as described elsewhere [8], [9]. Sequences were assessed for resistance associated mutations by entering them into the Stanford University HIV Drug Resistance Database [10].

### Determination of test characteristics

For serial dilution testing, amplifications were performed in triplicate and repeated, and detectable PCR product was evaluated at each input viral load level. For the patient samples, we determined the sensitivity, specificity, and positive and negative predictive values for the first- and second-round PCR reactions to detect varying levels of viremia (50, 100, 200, 300, 400, 500, 750 and 1,000 copies/mL) constituting the minipools based on previous viral load testing of individual samples. Cost savings were estimated based on the efficiency of the assay at each threshold and the cost of viral load assays and drug resistance testing at University of California, San Diego.

## Results

For the serial dilutions of RNA in spiked HIV-negative plasma, first-round PCR product was detected for all replicates with viral loads of 5,000 and 1,000 HIV RNA copies/mL. For patient samples, 295 blood plasma samples from 171 patients were included for 295 unique patient visits. The majority (95%) of the cohort was male, with a mean age of 37 years (range 21–62 years), and most (90%) were men who have sex with men (see **Table 1**). Based on previous individual quantitative testing, 43 of 295 samples (15%) had detectable HIV RNA (≥50 copies/mL), including four (1%) with viral loads of 1,000 copies/mL or greater. Among detectable samples, the viral load ranged from 50 to 10,500 copies/mL, with a median viral load of 231 copies/mL (see **Table 2**).

**Table 1:** Patient demographic data (for n = 171 patients).

Demographic	Total
**Male sex, n (%)**	162 (95)
**Age, mean in years (range)** §	37 (21–62)
**Ethnicity, n (%):**	
White	124 (73)
African American	8 (5)
Latino/a	30 (18)
Asian	6 (4)
Other	3 (2)
**HIV Risk Factor, n (%)**	
MSM	154 (90)
MSM+IDU	3 (2)
Heterosexual	12 (7)
Other	2 (1)

MSM = men who have sex with men; IDU = injection drug users.

§ Age was determined at the time of acquisition of the first chronological sample collected from an individual patient that was included in the analysis.

**Table 2:** Treatment and laboratory data for samples (n = 295)*.

Variable	Total
**ART regimen**	
PI based, n (%)	138 (47)
NNRTI based, n (%)	128 (43)
PI and NNRTI based, n (%)	9 (3)
NRTI only, n (%)	20 (7)
**CD4 cell counts at sample acquisition**	
Median CD4 cell count, median/µL (range)	656 (152–1415)
CD4<200 cells/µL, n (%)	1 (0)
CD4 200–350 cells/µL, n (%)	17 (6)
CD4 350–500 cells/µL, n (%)	52 (18)
CD4>500 cells/µL, n (%)	225 (76)
**Viral load** &	
Median detectable viral load, copies/mL (range)	231 (50–10,500)
VL<50 copies/mL, n (%)	252 (85)
VL 50–100 copies/mL, n (%)	12 (4)
VL 101–500 copies/mL, n (%)	21 (7)
VL 501–1,000 copies/mL, n (%)	6 (2)
VL>1,000 copies/mL, n (%)	4 (1)

ART = antiretroviral therapy, PI = protease inhibitor, NNRTI = non-nucleoside reverse transcriptase inhibitor, NRTI = nucleoside reverse transcriptase inhibitor, VL = viral load.

* Ninety-one (out of 171) patients are represented with multiple samples (maximum number of samples per patient = 3).

& For three samples with missing viral load information values were imputed as <50 copies/mL based on chronologically close viral load information.

**Figure 1:**
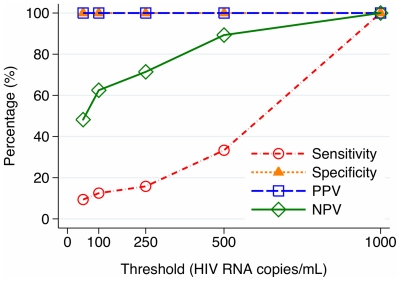
Test characteristics of qualitative pooled RT assay in the detection of varying levels of virologic failure using first round of PCR only. PPV = positive predictive value, NPV = negative predictive value.

The qualitative pooled RT assay was performed on 59 minipools of 5 patient samples (N = 295 samples). Of these, 39 minipools yielded no PCR product after two rounds, 3 yielded product after the first round, and 17 yielded product only after both rounds. The assay detected individual samples with ≥1,000 HIV RNA copies/mL with 100% sensitivity using one or two rounds of PCR (four samples with 1390, 2038, 4782 and 10,500 copies/mL each). The sensitivities of the first- and second-round PCR were 9% and 56% to detect samples with ≥50 copies/mL and 33% and 89% to detect samples harboring ≥500 copies/mL. Negative predictive values were around 90% or higher for both rounds of PCR to detect ≥500 copies/mL. The test characteristics of the pooled RT assay to detect varying levels of virologic failure in individual samples using one and two rounds of PCR are displayed in **Figures 1** **and** **2**. Screening of these 295 samples for virologic failure with the pooled RT assay would require 159 assays, including pooled and deconvolution testing, compared to 295 assays with individual testing. The per-assay costs of viral load and pooled RT assays at UCSD were $97 and $11 (slightly less for only one round of PCR) respectively. Based on these assay costs, between $1,752 and $1,775 would have been spent to screen 295 samples for virologic failure using the pooled RT assay, compared to $28,662 for viral load testing using the Amplicor viral load assay.

**Figure 2:**
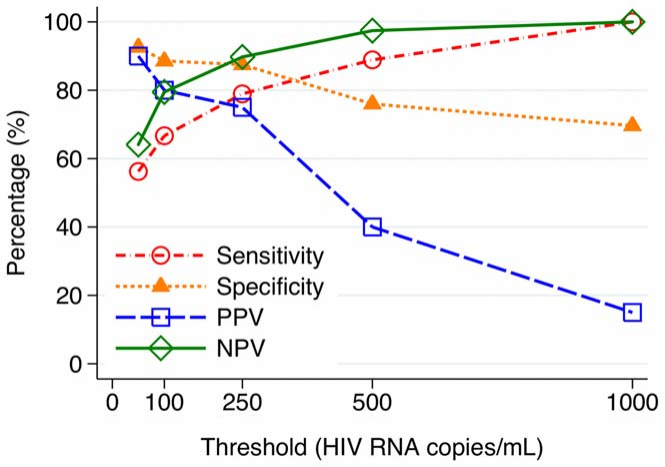
Test characteristics of qualitative pooled RT assay in the detection of varying levels of virologic failure using first and second rounds of PCR. PPV = positive predictive value, NPV = negative predictive value.

Sequence data for the HIV-1 RT coding region were successfully generated from each of the 20 positive minipools. Of these sequences (pools), six (30%) had mutations associated with low-, intermediate-, and/or high-level resistance to nucleoside or non-nucleoside reverse transcriptase inhibitors. The most common mutation was K103N, followed by mutations at positions 184 (M184MV, M184I) and 215 (T215CY, T215Y, T215D) of HIV-1 RT. The cost of sequencing amplified PCR product, including PCR clean-up, was $87 per sample, compared to around $300 for commercial genotyping. Therefore, the total cost of sequencing the PCR product of positive pools using the pooled RT assay would have been $1,739, compared to $6,000 for commercial genotyping.

## Discussion

Although quantitative viral load testing on pooled specimens has shown promise in reducing the cost of virologic monitoring of ART in resource-limited settings [2], [3], [4], [5], it is still expensive and does not provide information regarding drug resistance, which is clinically more useful than the viral load itself. This study presented a qualitative PCR-based platform that can detect levels of viremia at which true virologic failure is likely and at which the presence of drug resistance may be evaluated. Further, this new platform evaluated the RT coding region of the HIV-1 *pol* gene using primers that are conserved across clades, since the vast majority of first-line ART regimens in resource-limited settings are comprised of NNRTI and NRTIs. This allows for the PCR product to be sequenced for the presence of the mutations most likely to be associated with ART failure in these settings. This platform, however, can also be adapted to evaluate populations using protease inhibitor (PI) based therapy by adding an additional PCR assay using primer sets that cover the HIV-1 *pro* coding region.

Unlike quantitative pooling methods [2], [3], [4], [5], the qualitative nature of the new assay will require that all positive pools must be resolved by retesting of all individual samples in those pools using the same PCR assay [11]. Despite this, the pooled RT assay in this study was much more efficient than individual viral load testing, requiring just over half of the assays required for individual testing of the cohort. Although we did not perform deconvolution testing in real time, we were able to calculate the number of assays needed based on the pool positivity rate, since all samples in positive pools would be retested individually. Based on per-assay costs, screening of these samples using the pooled RT assay would have saved approximately $26,900 compared to individual viral load testing, and sequencing of the PCR product would have saved an additional $4,260 compared to commercial genotyping. Therefore, even in populations with higher rates of virologic failure for which relative efficiency of pooled testing would be lower in terms of the total number of assays needed, the cost of monitoring for virologic failure and drug resistance would still be significantly lower than that of commercial viral load testing and genotyping. For example, even in populations with virologic failure rates up to 30%, this assay would likely cost no more than 25% of standard methods. However, a full cost-effectiveness analysis was not performed in this retrospective study, and future studies should evaluate the real-time costs of assays, equipment maintenance, decontamination processes, and quality assurance measures in resource-limited settings to further evaluate feasibility of this method. Nevertheless, this potential cost savings in areas with limited second-line therapeutic options and high disease burden could then allow for more informed decisions regarding when to switch and which antiretroviral drugs are no longer efficacious.

Further, our platform detected all samples with ≥1,000 HIV RNA copies/mL with negative predictive values of 100%, even when only one round of PCR was performed, and requiring only one round of PCR would greatly decrease the complexity and cost of the assay. One round of PCR also offered an advantage over two rounds with respect to positive predictive value, although three pools with drug resistance mutations were only identified after two (not after one) round of PCR. Due to the qualitative nature of the assay, it was not possible to differentiate between samples that had lower and higher viral loads. At higher threshold levels for virologic failure, the false positive rate increased (for round two) due to the detection of samples with detectable viral loads that were below the set threshold for virologic failure. Therefore, the positive predictive value of the assay decreased as the threshold for virologic failure was raised when both rounds of PCR were performed. Since two rounds of PCR did not offer an advantage over one round for the detection of ≥1,000 HIV RNA copies/mL and had a higher false positive rate, we hypothesize that optimization of both assay sensitivity and positive predictive value will be achieved more easily using one round of PCR, although this should be validated in larger clinical cohorts. On one hand, the use of pools contributes to a higher false positive rate, but on the other hand it contributes to higher efficiency and lower costs. The impact of each should be investigated further in settings with a spectrum of different prevalence of virologic failure.

Concerning the detection of HIV-1 drug resistance, the PCR products of all of the positive minipools were successfully sequenced, and approximately one third of these harbored one or more mutations conferring some degree of resistance to reverse transcriptase inhibitors in one or both classes. When used in real time, sequencing would be performed on the PCR product of individual samples after deconvolution in order to give an individualized account of virologic failure and drug resistance profiles. Of course, these methods require laboratory settings with technical expertise in PCR and sequencing, which already exist in many resource-limited settings but are not used outside of the research setting due to cost. This combined lower-cost method would, therefore, allow for virologic monitoring and drug resistance genotyping to be made available for routine patient care in many of these areas and allow more informed decisions regarding when to change ART.

A major limitation of this study was a low prevalence of virologic failure among the study patients, of whom only four had viral loads above 1,000 copies/mL. Since the objective of this platform is to screen for levels of viremia likely to represent true ART failure (≥1,000 copies/mL), further evaluation using more samples with viral loads within the range of interest is needed to define the true sensitivity of the assay. Although the presented data indicate that the sensitivity of this assay is close to the desired target of at least one individual sample in the pool with 1,000 HIV RNA copies/mL using only one round of PCR, this protocol may require further optimization. For example, if the first-round PCR does not prove effective in detecting pools with at least one sample with ≥1,000 copies/mL in future applications, both rounds of PCR may be used to screen the minipools. Alternatively, if the assay is too sensitive, additional dilution with phosphate-buffered saline or another agent may be performed after pooling to achieve the desired sensitivity. Pool size may also be increased to lower sensitivity and cost, or a combination of increased pool size and further dilutions may be employed. The utility of these changes would mostly depend upon local factors, such as the prevalence of virologic failure in the population, the definition of virologic failure used by national ART committees and policymakers, and the number and frequency of samples screened. In addition, this study used banked specimens with different storage times, and there is the potential for RNA degradation in some samples that may have affected the results, and screening in real-time would be helpful to determine if this assay has the desired sensitivity or requires further optimization. Finally, since this study was performed using samples from a U.S. cohort, no patients infected with non-B subtypes were included in the analysis. Although the PCR primers used in this platform span a region in the RT coding region that is conserved across clades, future experiments should focus on the predominant infecting subtypes in the resource-limited settings that may benefit from the proposed methods.

In conclusion, we present a platform that incorporates sample pooling, PCR of the HIV-1 RT coding region and sequencing of generated PCR product that can be used to screen for both virologic failure of ART and drug resistance. Although further optimization of assay sensitivity and evaluation on non-B HIV-1 subtypes are needed, the lower assay costs and ability to combine virologic monitoring with drug resistance testing make it an attractive alternative to commercial assays. If proven effective, these methods would allow for maximization of first-line ART and decrease complications associated with delayed detection of ART failure in areas with limited resources for HIV services.

## Supporting Information

Figure S1 **CI_POL1 alignment with reference sequences for HIV-1 subtypes (alignments obtained using Los Alamos National Laboratory HIV Sequence Database,** http://www.hiv.lanl.gov **).**
(DOCX)

Figure S2 **3RT alignment with reference sequences for HIV-1 subtypes (alignments obtained using Los Alamos National Laboratory HIV Sequence Database,** http://www.hiv.lanl.gov **).**
(DOCX)

Figure S3 **5RT alignment with reference sequences for HIV-1 subtypes (alignments obtained using Los Alamos National Laboratory HIV Sequence Database,** http://www.hiv.lanl.gov **).**
(DOCX)
