# Supplementary material for: A Combined Screening Platform for HIV Treatment Failure and Resistance
Source: PLoS One. 2012 Apr 26;7(4):e35401. doi: 10.1371/journal.pone.0035401 (PMC3338506; doi:10.1371/journal.pone.0035401)
Supplement: Figure S2 — 3RT alignment with reference sequences for HIV-1 subtypes (alignments obtained using Los Alamos National Laboratory HIV Sequence Database, http://www.hiv.lanl.gov ). (DOCX) [file pone.0035401.s002.docx]

10 20

....|....|....|....|....|

**3RT**  **AGAACCTCCATTCCTTTGGATGGGT**

**B.US.1990.WEAU160_GHOSH.U21135** **...C.....................**

**G.KE.1993.HH8793_1_1.AF061640**  **........................A**

**D.CM.2001.01CM_4412HAL.AY37115** **............T............**

**B.NL.2000.671_00T36.AY423387**  **......C..................**

**B.US.1998.15384_1.DQ853463**  **.........................**

**H.CF.1990.056.AF005496**  **......C.....T...........A**

**H.BE.1993.VI997.AF190128**  **......C.....T...........A**

**B.FR.1983.HXB2-LAI-IIIB-BRU.K0** **.........................**

**C.BW.1996.96BW17.AF110980**  **......C.....T...........G**

**J.SE.1994.SE9173_7022.AF082395** **............T...........A**

**G.BE.1996.DRCBL.AF084936**  **............T...........A**

**A1.SE.1994.SE7253.AF069670**  **............T...........A**

**H.BE.1993.VI991.AF190127**  **......C.....T...........A**

**A1.KE.1994.Q23_17.AF004885**  **........................A**

**A1.UG.1985.U455_U455A.M62320**  **............T...........G**

**J.SE.1993.SE9280_7887.AF082394** **............T...........A**

**C.BW.1996.96BW0502.AF110967**  **......C.....T...........G**

**C.BR.1992.BR025-d.U52953**  **......C.....T...........G**

**D.CD.1984.84ZR085.U88822**  **............T............**

**G.SE.1993.SE6165_G6165.AF06164** **........................A**

**G.KE.1993.HH8793_12_1.AF061641** **........................A**

**D.TZ.2001.A280.AY253311**  **............T............**

**A1.UG.1992.92UG037.U51190**  **............T...........A**

**F1.FI.1993.FIN9363.AF075703**  **...............C........G**

**D.CD.1983.ELI.K03454**  **......C.....T............**

**A1.UG.1992.92UG037.AB253429**  **............T...........A**

**D.UG.1994.94UG114.U88824**  **......C.....T............**

**G.NG.1992.92NG083_JV10832.U888** **........................A**

**C.IN.1995.95IN21068.AF067155**  **......C.....T...........G**

**B.US.1998.1058_11.AY331295**  **........................G**

**A2.CY.1994.94CY017_41.AF286237** **............T...........A**

**D.CD.1983.NDK.M27323**  **............T............**

**F2.CM.1995.95CM-MP255.AJ249236** **......C.....T...........G**

**B.TH.1990.BK132.AY173951**  **.........................**

**B.US.1986.JRFL_JR_FL.U63632**  **.........................**

**K.CD.1997.97ZR-EQTB11.AJ249235** **......C.....T...........G**

**A1.AU.2003.PS1044_Day0.DQ67687** **......G.....T...........A**

**J.CD.1997.J_97DC_KTB147.EF6141** **......C.....T...........A**

**A1.RW.1992.92RW008.AB253421**  **........................A**

**C.ZA.2004.04ZASK146.AY772699**  **......C.....T...........G**

**B.US.1983.RF_HAT3.M17451**  **............T............**

**F2.CM.2002.02CM_0016BBY.AY3711** **G.....C.....T...........G**

**G.PT.-.PT2695.AY612637**  **........................A**

**F2.CM.1997.CM53657.AF377956**  **G.....C.....T...........G**

**F1.BR.1993.93BR020_1.AF005494**  **......C.................G**

**F1.BE.1993.VI850.AF077336**  **........................G**

**K.CM.1996.96CM-MP535.AJ249239**  **......C.....T...........A**

**A2.CD.1997.97CDKTB48.AF286238**  **............T..G........A**

**C.ET.1986.ETH2220.U46016**  **............T...........G**

**F1.FR.1996.96FR-MP411.AJ249238** **......C.................G**

**F2.CM.1995.95CM-MP257.AJ249237** **......C.....T...........G**

**A1.UG.1998.98UG57136.AF484509**  **......C.....T...........A**
